# Recurrence/prognosis estimation using a molecularly positive surgical margin‐based model calls for alternative curative strategies in pIIIA/N2 NSCLC

**DOI:** 10.1002/1878-0261.13600

**Published:** 2024-02-07

**Authors:** Li Li, Kewen He, Tao Zhou, Yang Xu, Jiaohui Pang, Qingxi Yu, Yongsheng Gao, Hongjin Shi, He Zhu, Mengke Li, Jinming Yu, Shuanghu Yuan

**Affiliations:** ^1^ Department of Radiation Oncology, Shandong Cancer Hospital and Institute Shandong First Medical University and Shandong Academy of Medical Sciences Jinan China; ^2^ Geneseeq Research Institute Nanjing Geneseeq Technology Inc. China; ^3^ Department of Pathology, Shandong Cancer Hospital and Institute Shandong First Medical University and Shandong Academy of Medical Sciences Jinan China; ^4^ Department of Radiation Oncology and Shandong Provincial Key Laboratory of Radiation Oncology, Shandong Cancer Hospital and Institute Shandong First Medical University and Shandong Academy of Medical Sciences Jinan China; ^5^ Research Unit of Radiation Oncology Chinese Academy of Medical Sciences Jinan China; ^6^ Department of Radiation Oncology, The First Affiliated Hospital of USTC, Division of Life Sciences and Medicine University of Science and Technology of China Hefei China

**Keywords:** COX model, metastatic lymph node ratio, molecularly positive surgical margin, next‐generation sequencing, non‐small cell lung cancer

## Abstract

Stage pIIIA/N2 non‐small cell lung cancer (NSCLC) is primarily treated by complete surgical resection combined with neoadjuvant/adjuvant therapies. However, up to 40% of patients experience tumor recurrence. Here, we studied 119 stage pIIIA/N2 NSCLC patients who received complete surgery plus adjuvant chemotherapy (CT) or chemoradiotherapy (CRT). The paired tumor and resection margin samples were analyzed using next‐generation sequencing (NGS). Although all patients were classified as negative resection margins by histologic methods, NGS revealed that 47.1% of them had molecularly positive surgical margins. Patients who tested positive for NGS‐detected residual tumors had significantly shorter disease‐free survival (DFS) (*P* = 0.002). Additionally, metastatic lymph node ratio, erb‐b2 receptor tyrosine kinase 2 (*ERBB2*) mutations, and SWI/SNF‐related, matrix‐associated, actin‐dependent regulator of chromatin, subfamily a, member 4 (*SMARCA4*) mutations were also independently associated with DFS. We used these four features to construct a COX model that could effectively estimate recurrence risk and prognosis. Notably, mutational profiling through broad‐panel NGS could more sensitively detect residual tumors than the conventional histologic methods. Adjuvant CT and adjuvant CRT exhibited no significant difference in eliminating locoregional recurrence risk for stage pIIIA/N2 NSCLC patients with molecularly positive surgical margins.

## Graphical abstract

Tumor‐derived mutations were detected in 47.1% of histologically negative resection margins collected from 119 stage pIIIA/N2 non‐small‐cell lung cancer (NSCLC) patients through next‐generation sequencing (NGS). The COX model generated by using four clinical/molecular features, including NGS‐detected positive resection margin, metastatic lymph node ratio, *ERBB2* mutations, and *SMARCA4* mutations, could effectively estimate disease relapse and prognosis of patients following curative‐intent surgery.

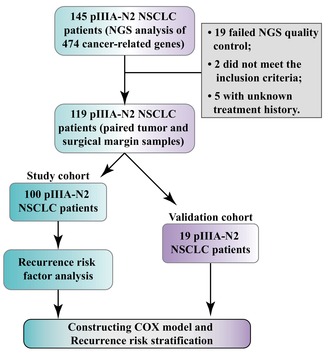

**Abbreviations**
ADC: adenocarcinoma
CNA: copy number alteration
CNV: copy number variant
CRT: chemoradiotherapy
CT: chemotherapy
DFS: disease‐free survival
ExAC: exome aggregation consortium
FFPE: formalin‐fixed paraffin‐embedded
GATK: genome analysis toolkit
H&E: hematoxylin and eosin
KPS: Karnofsky performance status
mDFS: median disease‐free survival
MLNR: metastatic lymph node ratio
mOS: median overall survival
MRD: minimal residual disease
NCCN: National Comprehensive Cancer Network
NGS: next‐generation sequencing
NSCLC: non‐small cell lung cancer
OS: overall survival
PORT: postoperative radiotherapy
SCC: squamous cell carcinomas
SNP: single nucleotide polymorphism
SNV: single nucleotide variant
SV: structural variant
VAF: variant allele frequency

## 1 Introduction

Around one‐third of non‐small cell lung cancer (NSCLC) patients have stage III diseases at initial diagnosis, and stage pIIIA/N2 NSCLC is characterized by relatively small tumors and ipsilateral and/or subcarinal mediastinal lymphatic spread [1, 2]. Various clinical and retrospective studies have been conducted to explore the ideal strategy in stage pIIIA/N2 patients, with reported 5‐year survival rates ranging from 15% to 50% [3, 4, 5, 6]. For resectable stage pIIIA/N2 NSCLC, complete surgical resection (R0 resection) combined with various neoadjuvant/adjuvant therapies was the main treatment mode [3, 7]. The adjuvant chemotherapy was considered to improve survival in stage pIIIA/N2 NSCLC patients treated with R0 resection. Although postoperative radiotherapy (PORT) failed to confer overall disease‐free survival (DFS) and overall survival (OS) benefits to stage pIIIA/N2 NSCLC patients, it could help control the locoregional disease [3, 8, 9]. Notably, despite the lower local recurrence rate in PORT‐treatment patients compared with that in control patients, there were still a considerable number of patients suffering from local disease relapse. The underlying rationale for including radiotherapy in the adjuvant treatment regimen is that radiotherapy could effectively eliminate the residual cancer cells near the surgical site. However, in cases where there are no residual tumors, the toxic side effects of radiotherapy outweigh its therapeutic values, which may lead to radiotoxicity‐related death and compromise the overall benefits of radiotherapy on DPS and OS. As a result, it is crucial to identify patients with postoperative minimal residual disease (MRD) before applying adjuvant radiotherapy.

In curative‐intent surgeries, it is imperative to achieve R0 resection, that is, there is no macroscopic and microscopic level of malignancy at the resection margin [10]. In the case of microscopic (R1) or macroscopic (R2) levels of residual diseases, patients were often associated with a worse prognosis and increased risk of recurrence [11, 12, 13, 14]. However, despite R0 resection, as high as 40% of stage pIIIA/N2 NSCLC patients suffered from local recurrence or regional lymph node metastasis within 5 years of initial operation [15]. Given the controversial benefits of PORT in stage pIIIA/N2 patients, we speculated that some stage pIIIA/N2 patients might have microscopically undetectable residual tumors after the systematic therapy suggested by the National Comprehensive Cancer Network (NCCN) guidelines [16]. With the development of molecular diagnostic approaches, particularly next‐generation sequencing (NGS), it is feasible to evaluate the malignant potential of surgery margin at molecular levels. Also, it is of clinical importance to identify prognostic factors and stratify the risk of recurrence for stage pIIIA/N2 NSCLC.

In this study, we retrospectively analyzed 119 stage pIIIA/N2 NSCLC patients who received complete surgery (R0 resection) plus adjuvant chemotherapy (CT) or chemoradiotherapy (CRT). Broad‐panel NGS was performed on the paired tumor and resection margin samples from the 119 patients, and the associations among the molecular level at the resection margin, clinical/molecular features, adjuvant treatment strategy, and clinical outcomes were then explored.

## 2 Materials and methods

### 2.1 Patients and study design

**Fig. 1:**
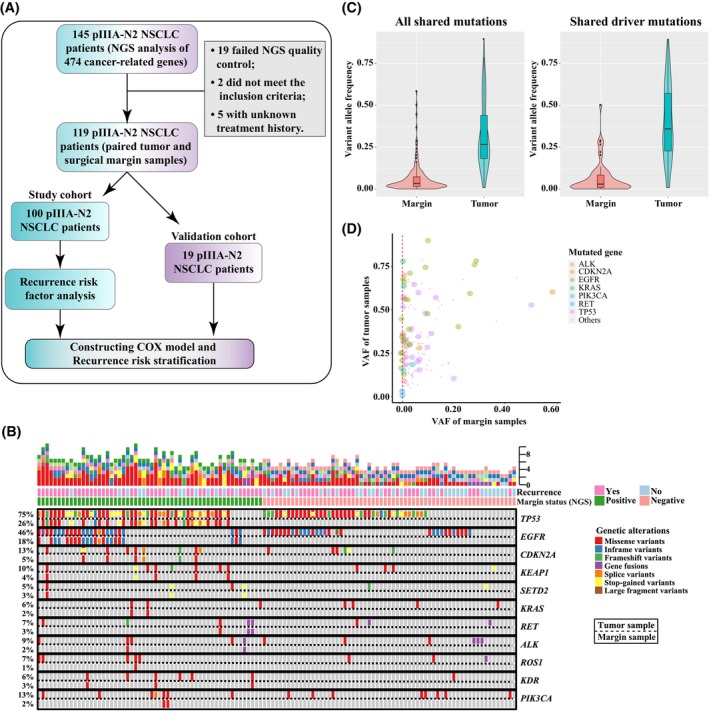
Detection of a considerable amount of lung cancer driver mutations in surgical resection margin. (A) The workflow of the study design. (B) The genetic profile of the paired tumor and resection margin samples of the 119 stage pIIIA/N2 NSCLC patients. The frequency of each genetic alteration was labeled on the left and the type of genetic alterations were listed on the right of the oncoprint fig. (C) The violin plot of VAF for all shared mutations or shared lung cancer driver mutations in resection margin and tumor samples. (D) The bubble plot VAF of tumor samples versus VAF of resection margin samples. Each dot represented a mutation that was simultaneously detected in the paired tumor and resection margin samples of a specific patient in the study group. NGS, next‐generation sequencing; pIIIA, pathologic stage IIIA; VAF, variant allele frequency.

This study was conducted in accordance with the declaration of Helsinki and was approved by Shandong Cancer Hospital and Institute (ethical number: SDZLEC201804301). All patients provided written informed consent to participate and publication. A total of 145 NSCLC patients who were initially diagnosed with pathologic stage pIIIA/N2 disease at Shandong Cancer Hospital and Institute from September 2014 to November 2018 were included in this retrospective cohort study (Fig. 1A). All patients were above 18 years old at the time of treatment and had a Karnofsky Performance Status (KPS) of ≥ 70. Only patients with R0 resections, which were confirmed by hematoxylin and eosin (H&E) and light microscopy techniques, were included. Samples from 19 patients did not pass the sequencing quality control, 1 patient received preoperative neoadjuvant chemotherapy, 1 patient had concomitant hypopharyngeal cancer, and 5 patients had unknown post‐surgical treatment history; these 26 patients were, thereby, excluded from further analyses. The remaining 119 patients had paired tumor and resection margin samples collected during complete surgical resection. Margin tissues were collected at the bronchial resection margin, with a minimum distance of 2.0 centimeters (cm) from the tumor site per the recommendations provided by the National Comprehensive Cancer Network guidelines [17, 18, 19]. For both the tumor tissue and paired bronchial resection margin tissue, the same formalin‐fixed paraffin‐embedded (FFPE) block was consecutively sliced for the evaluation of tumor cell content (TCC) by histologic H&E staining and mutational profiling using a pan‐cancer NGS panel covering 474 cancer‐relevant genes, including genes related to the efficacy and toxicity of radiotherapy/chemotherapy, the target genes of classic targeted therapies, the immunotherapy‐associated genes, as well as genes related to tumorigenesis and familial cancers (Table S1). All the bronchial resection margins were classified as tumor‐negative using the conventional histologic H&E staining, and the results were verified by two experienced pathologists. To study the recurrence risk in stage pIIIA/N2 NSCLC, we analyzed data using 100 patients in the study cohort and subsequently conducted validation using data from an additional 19 patients (Fig. 1A). It was worth mentioning that, despite an anticipated patient enrollment of 100 patients for both the study and the validation cohorts, only 19 patients who were consecutively enrolled at the participating hospital between September 2014 and November 2018 fulfilled the inclusion criteria and were included in the validation cohort.

### 2.2 DNA extraction and library preparation

DNA extraction and sequencing library construction were carried out following standard protocols as previously described [20]. In brief, FFPE tumor and resection margin samples were de‐paraffinized with xylene, and genomic DNA was extracted using the QIAamp DNA FFPE Tissue Kit (Qiagen, Hilden, Germany). Purified genomic DNA was qualified by Nanodrop2000 for A260/A280 and A260/A230 ratios (Thermo Fisher Scientific, Waltham, MA, USA). All DNA samples were quantified by Qubit 3.0 using the dsDNA HS Assay Kit (Life Technologies, Carlsbad, CA, USA) according to the manufacturer's recommendations.

Sequencing libraries were prepared using the KAPA Hyper Prep kit (KAPA Biosystems, Wilmington, MA, USA) with an optimized manufacturer's protocol [20]. Briefly, approximately 1–2 μg of DNA underwent sequential processes of end‐repair, A‐tailing, and ligation with indexed adapters. This was followed by size selection using Agencourt AMPure XP beads (Beckman Coulter, Mississauga, ON, Canada) and PCR amplification using the KAPA Hyper DNA Library Prep Kit (KAPA Biosystems). Target enrichment was performed using customized xGen lockdown probes (Integrated DNA Technologies, Coralville, IA, USA) targeting 474 cancer‐relevant genes (Radiotron^®^, Nanjing Geneseeq Technology Inc., Nanjing, China). The hybridization capture reaction was performed with Dynabeads M‐279 (Life Technologies) and xGen Lockdown hybridization and wash kit (Integrated DNA Technologies) according to the manufacturer's protocols. The captured libraries were on‐beads PCR amplified with Illumina p5 and p7 primers in KAPA HiFi HotStart ReadyMix (KAPA Biosystems), followed by purification using Agencourt AMPure XP beads. Libraries were quantified by qPCR using the KAPA Library Quantification Kit (KAPA Biosystems). Library fragment size was determined by Bioanalyzer 2100 (Agilent Technologies, Waldbronn, Germany).

### 2.3 Next‐generation sequencing and data processing

Sequencing was performed on the Illumina HiSeq4000 platform followed by data analysis as previously described [20, 21]. In brief, sequencing data were analyzed by Trimmomatic [22] to remove low‐quality (quality < 15) or N bases, and then mapped to the human reference genome hg19 using the Burrows‐Wheeler Aligner (https://github.com/lh3/bwa/tree/master/bwakit). PCR duplicates were removed by Picard (https://broadinstitute.github.io/picard/). The Genome Analysis Toolkit (GATK) (https://software.broadinstitute.org/gatk/) was used to perform local realignments around indels and base quality reassurance. Single nucleotide polymorphisms (SNPs) and indels were analyzed by varscan2 [23] and Haplotype Caller in GATK. Common SNPs were excluded if they were present in > 1% population frequency in the 1000 Genomes Project or the Exome Aggregation Consortium (ExAC) 65 000 exomes database. Gene fusions were identified by FACTERA [24]. The in‐house developed bioinformatics pipeline has been previously used and reported by others [25, 26].

The cut‐offs of variant allele frequency (VAF) and sequencing reads/depth for tumor samples were as follows: VAF ≥ 0.5%, supporting reads ≥ 3, depth ≥ 30× for recurrent variants (≥ 20 mentions in COSMIC v92 [27]), and VAF ≥ 1%, supporting reads ≥ 6, and depth ≥ 30× for non‐recurrent variants; structural variant (SV) was split‐reads ≥ 3; copy number variants (CNVs) were hotspots in TCGA database [28] with gene ratio ≥ 2 as copy number gain and gene ratio < 0 .6 as copy number loss. The mutation calling thresholds used for resection margin samples were as follows: recurrent variants were VAF ≥ 0.5% or supporting reads ≥ 3 and depth ≥ 30×; non‐recurrent variants were VAF ≥ 1% or supporting reads ≥ 6 and depth ≥ 30×; structural variant (SV) was split‐reads ≥ 3. To note, the NGS‐based surgical margin positivity was defined by the presence of one or more genetic variants that were also identified in the matched tumor sample.

### 2.4 Mutation signature analysis

Samples with synonymous/non‐synonymous mutations of ≥ 5 were included for mutation signature analysis [29], which was conducted using the “maftools” and “sigminer” r packages. Based on the description of the 30 mutational signatures listed on the COSMIC website (https://cancer.sanger.ac.uk/signatures/signatures_v2/), we classified the signatures into 10 groups (Table S2).

### 2.5 Statistical analysis

Disease‐free survival (DFS) refers to the time from the initiation of curative‐intent surgery to tumor relapses/patient death, and overall survival (OS) refers to the time from the initial diagnosis to patient death. For loss to follow‐up patients, their OS was calculated based on the time of the last follow‐up. Kaplan–Meier survival curve was used to analyze the DFS or OS of various patient groups, and the statistical difference was analyzed using the log‐rank test. Univariate and multivariate COX analyses were performed to evaluate the predictive values of various pathological and molecular features on DFS. The correlation analysis of dichotomous variables was conducted by the φ‐Phi coefficient for non‐normally distributed data, such as the Wilcoxon test for mutational signature analysis. Statistical analyses were performed using the r (v4.1.0), and a two‐sided *P* value of < 0.05 (*P < 0.05, **P < 0.01, ***P < 0.001) was considered to be statistically significant.

## 3 Results

### 3.1 Patient characteristics

The clinical characteristics of the 119 stage pIIIA/N2 NSCLC patients are summarized in Table 1 and Fig. S1. Specifically, the median age of the patients was 60 years, and 64.7% of them were males. Around half of the patients (51.3%) did not have a smoking history. The majority of the patients had either adenocarcinoma (ADC; 58.8%) or squamous cell carcinomas (SCC; 31.9%), with 63.0% of the tumors located on the right side of the lung. The median KPS was 90, and all patients received R0 resection followed by adjuvant therapy CT (*n* = 84; 70.6%) or CRT (*n* = 35; 29.4%). Except for three patients who received pneumonectomy, the remaining 116 patients had either lobectomy or segmentectomy. According to the H&E staining approach, all of the resection margin samples from the 119 patients did not have detectable residual tumor cells (i.e., R0‐resection). The median metastatic lymph node ratio (MLNR) was 23.1%, with the median number of examined lymph nodes of 17 and the median number of positive lymph nodes of 4.

**Table 1:** The clinical characteristics of the 119 pIIIA/N2 NSCLC patients who received complete surgical resection.

Characteristics	Individual (*n* = 119)	Percentage
Median follow‐up (months) [median (95% CI)]	37.4 (34.0~46.8)	
Median age (years) [median (range)]	60 (39~77)	
Sex		
Female	42	35.3
Male	77	64.7
Smoking history		
Yes	58	48.7
No	61	51.3
Pathological type		
Adenocarcinoma	70	58.8
Squamous cell carcinoma	38	31.9
Others	11	9.2
Operation procedure		
Pneumonectomy	3	2.5
Lobectomy/segmentectomy	116	97.5
Adjuvant therapy		
Chemotherapy	84	70.6
Chemoradiotherapy	35	29.4
Tumor location		
Left‐sided	44	37.0
Right‐sided	75	63.0
Resection margin status (H&E)		
Positive	0	0
Negative	119	100
Resection margin status (NGS)		
Positive	56	47.1
Negative	63	52.9
Performance status (KPS) [median (range)]	90 (60~100)	
Number of examined lymph nodes [median (range)]	17 (4~53)	
Number of positive lymph nodes [median (range)]	4 (1~23)	
Metastatic lymph node ratio (MLNR) [median (range)]	23.1% (1.9%~100.0%)	

### 3.2 NGS detected tumor mutation‐positive resection margins in microscopically tumor‐negative patients

We analyzed the resected tumor samples from the 119 patients using the broad‐panel NGS, and 98.3% of the tumors had detectable somatic genetic alterations, including 914 single nucleotide variants (SNVs), 142 indels, 101 copy number alterations (CNAs), and 26 gene fusions (Table S3). The most frequently mutated genes in tumors were *TP53*, *EGFR*, *CDKN2A*, *PIK3CA*, and *LRP1B* (Fig. S2A).

Strikingly, by analyzing the paired resection margin samples from the 119 patients using the same broad‐panel NGS, 56 (47.1%) resection margin samples were positive for tumor‐derived alterations, despite all of the resection margin samples being classified as tumor‐negative by the conventional H&E staining approach (Table 1). Indeed, a total of 247 somatic genetic alterations were detected in resection margin samples, including 209 SNVs, 33 indels, and 5 gene fusions (Table S4). The genetic profile of the resection margin samples was similar to that of tumor samples, although the mutational frequency was much lower (Fig. 1B and Fig. S2B). In addition, we found that the overall variant allele frequency (VAF) was significantly higher in tumor samples than in resection margin samples (Fig. 1C). For some patients, their tumor and resection margin samples had comparable VAF in certain mutated genes, most of which were lung cancer driver mutations, such as *EGFR* and *TP53* mutations (Fig. 1D and Fig. S2C). Furthermore, we compared genetic variants found in paired samples obtained from all 119 patients. Notably, most of the genetic variants exclusively identified in bronchial resection margin samples are of unknown clinical significance, with only *PIK3CA*‐H1047R in one patient being identified as a driver mutation according to the OncoKB classification (Table S5). Based on the tumor‐informed mutation calling method, 47.1% (56/119) of the margin samples tested positive for tumor mutations by NGS, with 47 of them (39.5%, 47/119) containing driver alterations (Table S6). The agreement of detection for driver alteration‐positive patients between tumor and matched margin samples was 41.6% (47/113). Overall, a considerable proportion of stage pIIIA/N2 patients, who were classified as tumor‐negative by the histologic method, exhibited molecularly positive surgical margins determined by NGS. Although we diligently followed the NCCN guidelines for a safe resection margin distance of 2.0 cm, and all bronchial resection margins were found to be free from tumor cells by the conventional method, it is evident that the examination of microscopically negative margins through NGS provides additional information and represents a valuable approach to identify molecular levels of residue tumors and to evaluate recurrence risk and estimate prognosis.

### 3.3 The NGS‐detected positive resection margin was associated with a higher recurrence risk

**Fig. 2:**
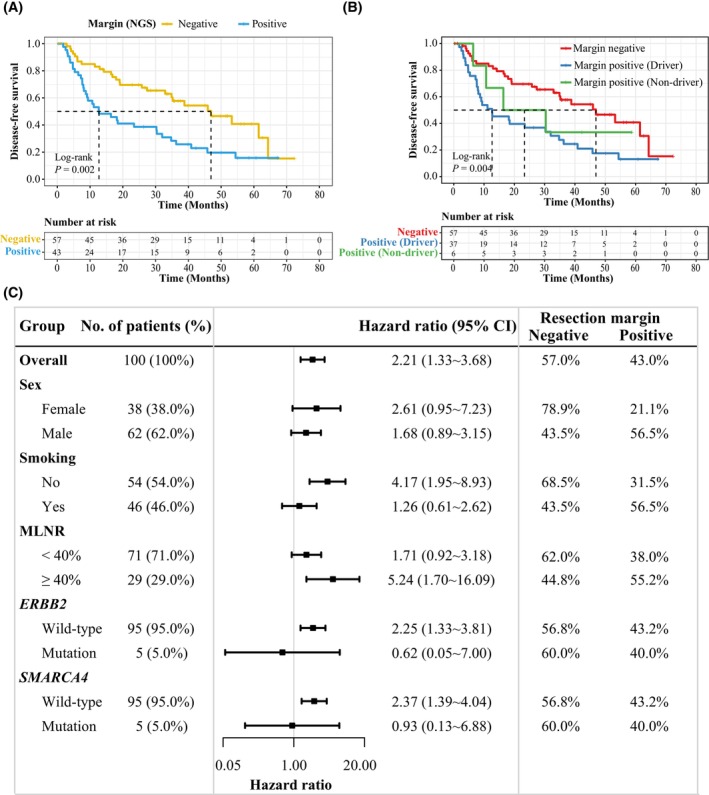
The presence of tumor mutations in resection margins was associated with shorter disease‐free survival. (A) Kaplan–Meier curve of disease‐free survival in 100 stage pIIIA/N2 NSCLC patients (study cohort) in strata of the presence or absence of tumor mutations in resection margin. The estimated median survival time is represented by the location on the x‐axis where the curve intersects with the horizontal dashed line drawn at a 50% survival probability. (B) Kaplan–Meier curve of disease‐free survival in 100 stage pIIIA/N2 NSCLC patients (study cohort) in strata of the resection margin mutational status (i.e., no mutations, with only non‐driver mutations, or with lung cancer driver mutations). The estimated median survival time is represented by the location on the *x*‐axis where the curve intersects with the horizontal dashed line drawn at a 50% survival probability. (C) The forest plot of the hazard ratio for DFS to analyze the effects of the NGS‐detected resection margin positivity after subgrouping of various clinical and molecular features in the study cohort. CI, confidence interval; DFS, disease‐free survival; No., number.

For all the 119 patients in our study, the median disease‐free survival (mDFS) and median overall survival (mOS) were 37.4 and 64.3 months, respectively, which was comparable to previous clinical studies [30, 31]. To study the recurrence risk in stage pIIIA/N2 NSCLC, we conducted survival analyses using both the study (*n* = 100) and validation (*n* = 19) cohorts. The clinical characteristics of the patients in these two cohorts are shown in Table S7. For the 100 patients in the study cohort (Fig. 1A), patients with NGS‐detected positive resection margin had significantly shorter DFS (*P* = 0.002, HR: 2.21, 95% CI: 1.33~3.68) than those with negative resection margin (Fig. 2A). By dividing margin mutations into lung cancer driver and non‐driver mutations, both patients with driver‐positive resection margins and patients with only non‐driver‐positive resection margins had worse DFS than those with NGS‐detected negative resection margin (*P* = 0.004) (Fig. 2B). Similar trends were observed when analyzing patients' overall survival in the study cohort (Fig. S3).

### 3.4 The recurrence risk analysis of stage pIIIA/N2 NSCLC

By performing the univariate COX analysis using various demographic and clinical features, we found that sex, smoking history, and metastatic lymph node ratio (MLNR) were significantly associated with disease relapse and patient prognosis (Fig. S4A,B, and Table 2). Additionally, we analyzed genes with mutated/CNV frequency higher than 5% (Table S8), and we found mutations of *SMARCA4*, *NOS2*, and *ERBB2* were significantly associated with worse DFS (*P* < 0.001, *P* = 0.009, and *P* = 0.042, respectively) and shorter OS (*P* = 0.020, *P* < 0.001, and *P* < 0.001, respectively) (Fig. S4C–H). We then conducted multivariate COX analysis using features that were significant in univariate COX analysis, and NGS‐detected positive resection margin (*P* = 0.042), MLNR (*P* = 0.041), *ERBB2* mutations (*P* = 0.035), and *SMARCA4* mutations (*P* = 0.021) remained to be statistically significant (Table 2). Similarly, the subgrouping analysis revealed that NGS‐detected positive resection margin appeared to be a profound hazard factor for disease recurrence after subgrouping patients based on various demographic, clinical, and molecular features (Fig. 2C), which further supported the independent association between NGS‐detected positive resection margin and the risk of tumor relapse.

**Table 2:** Univariate and multivariate COX analyses between demographic/clinical/molecular characteristics and disease‐free survival.

Characteristics	Univariate analysis		Multivariate analysis	
	HR (95% CI)	*P*	HR (95% CI)	*P*
Sex				
Male vs Female	2.26 (1.30~3.94)	0.003**		
Smoking history				
Yes vs No	1.67 (1.01~2.77)	0.045*	1.49 (0.79~2.78)	0.215
Metastatic lymph node ratio (MLNR)				
≥ 40% vs < 40%	1.72 (1.00~2.95)	0.046*	1.96 (1.03~3.72)	0.041*
Margin status (NGS)				
Positive vs Negative	2.21 (1.33~3.68)	0.002**	1.79 (1.02~3.13)	0.042*
*ERBB2*				
Mutation vs Others	2.81 (0.99~7.94)	0.042*	3.26 (1.08~9.77)	0.035*
*NOS2*				
Mutation vs Others	3.24 (1.27~8.25)	0.009**	1.49 (0.54~4.13)	0.447
*SMARCA4*				
Mutation vs Others	4.92 (1.89~12.78)	0.0003***	3.59 (1.22~10.56)	0.021*
*NFE2L2*				
Mutation vs Others	1.19 (0.47~2.97)	0.716		
*TP53*				
Mutation vs Others	1.86 (0.99~3.5)	0.051		
*LRP1B*				
Mutation vs Others	0.66 (0.3~1.47)	0.307		

### 3.5 Construction and validation of the COX model to stratify recurrence risk and estimate prognosis

**Fig. 3:**
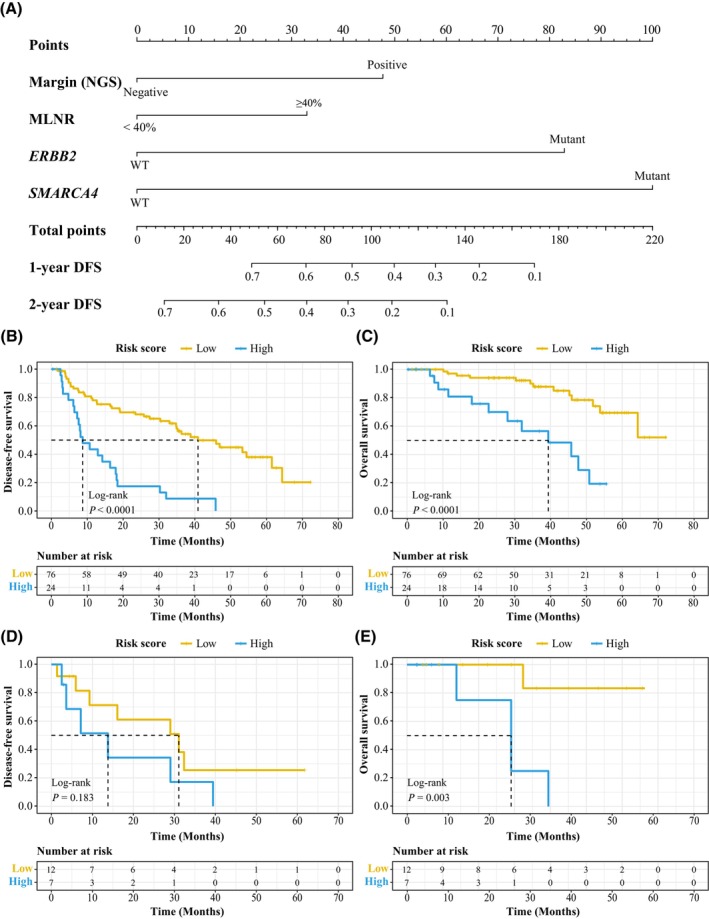
Constructing a COX model to predict the recurrence in stage pIIIA/N2 NSCLC patients. (A) The illustration of the nomogram of the COX proportional‐hazards model generated using NGS‐detect margin status, MLNR (≥ 40% vs < 40%), *ERBB2* mutation, and *SMARCA4* mutation. (B, C) Kaplan–Meier curve of DFS (B) or OS (C) in strata of COX model risk score in patients from the study cohort. The estimated median survival time is represented by the location on the x‐axis where the curve intersects with the horizontal dashed line drawn at a 50% survival probability. (D, E) Kaplan–Meier curve of DFS (D) or OS (E) in strata of COX model risk score in patients from the validation cohort. The estimated median survival time is represented by the location on the x‐axis where the curve intersects with the horizontal dashed line drawn at a 50% survival probability. DFS, disease‐free survival; MLNR, metastatic lymph node ratio; OS, overall survival.

As NGS‐detected positive resection margin, MLNR, *ERBB2* mutations, and *SMARCA4* mutations were independently associated with disease recurrence, we constructed a COX model using these 4 factors and demonstrated it using a nomogram (Fig. 3A). The 4‐factor COX model achieved an Akaike information criterion (AIC) of 466.09 and concordance index (C‐index) of 0.679 and removing any factor would result in an increase in AIC and a decrease in C‐index, especially the NGS‐detected positive resection margin (Table S9). As lower AIC and higher C‐index indicated a better fitting of the model, these results suggest that all four factors contributed to the estimation of tumor relapse in the study group. We also tested the model in the validation cohort, and the 4‐factor Cox model reached a comparable C‐index (Table S9).

Next, we separate patients into the high‐risk group and low‐risk group using the 4‐factor COX model, with the cut‐off being selected by X‐tile as described previously [32]. The low‐risk patients had a significantly longer DFS and OS than the high‐risk patients in the study cohort (*P* < 0.001; Fig. 3B,C), and a similar trend was observed in the validation cohort (*P* = 0.183 and *P* = 0.003, respectively; Fig. 3D,E), indicating that the 4‐factor Cox model could potentially estimate disease relapse and prognosis in stage pIIIA/N2 NSCLC patients who received curative‐intent surgery.

### 3.6 Mutational profiling by broad‐panel NGS was more sensitive than conventional histologic approaches to detect residue tumors

**Fig. 4:**
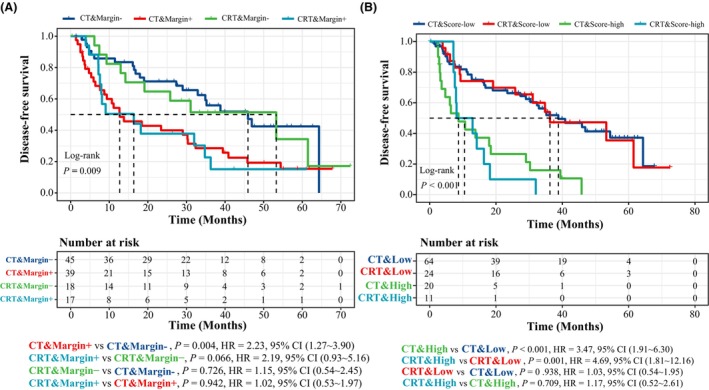
Adjuvant CRT did not provide additional benefits for patients' prognosis compared to adjuvant CT for patients with an NGS‐detected positive resection margin. (A) Kaplan–Meier curve of disease‐free survival in 119 stage pIIIA/N2 NSCLC patients in strata of the adjuvant treatment regimen and resection margin status. The estimated median survival time is represented by the location on the *x*‐axis where the curve intersects with the horizontal dashed line drawn at a 50% survival probability. (B) Kaplan–Meier curve of disease‐free survival in 119 stage pIIIA/N2 NSCLC patients in strata of the adjuvant treatment regimen and 4‐factor COX model risk scores. The estimated median survival time is represented by the location on the *x*‐axis where the curve intersects with the horizontal dashed line drawn at a 50% survival probability. CRT, chemoradiotherapy; CT, chemotherapy; NGS, next‐generation sequencing.

Lastly, we compared the clinical outcomes between the two treatment regimens of adjuvant therapies. Patients who received adjuvant CT had indistinguishable DFS and OS when compared with those who were treated with adjuvant CRT in our cohort (Fig. S5). Among patients with molecularly positive surgical margins, the inclusion of radiotherapy into the adjuvant CT regimen made no difference in terms of reducing recurrence risk (*P* = 0.942, HR: 1.02, 95% CI: 0.53~1.97, Fig. 4A). Surprisingly, the positivity of the resection margin itself could separate patients with a higher and lower probability of recurrence despite the adjuvant treatment strategy (Fig. 4A). A consistent trend was observed when separating high‐risk and low‐risk patients using the 4‐factor COX model (Fig. 4B). Overall, these findings suggest that adjusting the adjuvant treatment strategy from CT to CRT may not significantly eliminate the risk of recurrence for stage pIIIA/N2 NSCLC patients who have undergone complete surgical resection with detectable molecular abnormalities in their surgical margin tissues. Given that microscopically negative resection margins may exhibit molecular levels of residue tumors, our findings suggest that mutational analysis using NGS is a more sensitive approach compared to histological methods in identifying molecular abnormalities. Hence, NGS profiling of surgical margins following curative‐intent complete surgery should be considered as an additional valuable approach for assessing recurrence risk and estimating prognosis, ultimately enhancing disease management.

## 4 Discussion

Patients with stage pIIIA/N2 NSCLC usually suffer from poor disease control and low overall survival [33, 34, 35], so it is of clinical importance to develop reliable biomarkers to estimate the disease recurrence and prognosis for these patients. In the present research, we detected nearly half of the patients with H&E negative surgical margins have NGS‐detected positive resection margins, and these molecular levels of residual tumor cells were likely to contribute to significantly higher recurrence risk and poorer prognosis, even after considering other clinical and molecular risk factors, including smoking history, the number of positive lymph nodes, MLNR, and *KRAS* mutational status. We then generated a 4‐factor COX model, including NGS‐detected positive resection margin, MLNR, *ERBB2* mutations, and *SMARCA4* mutations, and demonstrated its promising capacity to estimate disease relapse and patient prognosis. Notably, adjuvant CRT and CT made no difference in reducing the locoregional recurrence risk of patients with molecularly positive surgical margins.

Stage pIIIA/N2 NSCLC patients with non‐bulky, discrete, or single‐level N2 involvement are recommended to receive R0 resection [16, 36], and the combination of surgical resection with other neoadjuvant/adjuvant CT could dramatically improve patient survival [37, 38, 39, 40]. Additionally, a large number of clinical studies have reported the value of PORT combined with clinical and anatomical factors [8, 9, 41, 42, 43, 44, 45], and the inconsistent results indicated that PORT could not be recommended indiscriminately in all pIIIA/N2 patients. Considering that patients with R0 resection still suffered from high local/distal recurrence [15, 46], the H&E‐based R0 resection may not accurately reflect the molecular biological status of individual resection margins. Molecular diagnostic approaches have been applied to assess surgical margins recently. Kim *et al*. [47] found that nearly 53% of the pancreatic cancer patients had detectable *KRAS* mutations in their H&E‐negative surgical margins samples, and these pancreatic cancer patients who were positive for surgical margins by the PCR‐based approach had a worse prognosis. Several gene mutations and DNA promoter hypermethylation changes were also successfully found in the surgical margins of NSCLC [48, 49]. However, these studies had limited cohort size, utilized single gene sequencing data, or mainly focused on stage I‐II patients.

Our study conducted a pioneering work of utilizing the broad‐panel NGS approach to assess the resection margin in 119 pIIIA/N2 NSCLC patients. Nearly 47% of them had molecular levels of MRD at the resection margin in 119 stage pIII/N2 patients with negative histologic resection margins, that is, retaining tumor residues at the molecular level. Generally, VAF correlates with the TCC assessed through light microscopy of H&E‐stained slides. Nonetheless, it is important to recognize that this assumption may not always prove valid. Here, we provide three putative explanations to illustrate why genetic variations being detected in morphologically negative samples are possible. Firstly, although we strictly adhered to instructions outlined in the NCCN guidelines for the collection of surgical margin tissues, it is worth noting that clear evidence is still lacking with respect to the most appropriate surgical margin for limited resections. Indeed, the TCC results that were reviewed by two experienced pathologists yielded negative H&E staining for all 119 surgical margin samples. Therefore, our findings suggest that NGS offers a valuable approach to detecting molecular levels of residue tumors in microscopically negative margins and evaluating recurrence risk and patient prognosis. Secondly, although slides employed for H&E staining and NGS were sequentially sliced from the same FFPE block, tumor heterogeneity, potentially resulting from sampling, may have led to disparities in TCC and VAF results. Lastly, there is a possibility of surgical margin tissues being putative cancerized fields that are composed of cells carrying some but not all phenotypes required for malignancy. Indeed, the concept of field cancerization, also known as field effects or field defects, was first proposed decades ago in 1953 [50]. Intriguingly, the affected tissue may appear histologically normal but carries genetic alterations that make it more prone to developing additional cancerous lesions [51]. However, it should be noted that putative cancerized fields are common but not all can progress to cancer. Hence, assuming that the detection of a cancerized field is indicative of probable cancer development poses the risk of overdiagnosis as well as overtreatment. Further research is warranted to conclusively ascertain whether the margin tissue represents cancerized fields, or whether these pre‐malignant sites have the potential to progress into cancer.

Interestingly, significantly decreased DFS and OS were observed in patients with positive over those with negative NGS‐detected resection margins regardless of adjuvant CT or CRT in patients with negative histologic resection margins. The H&E‐based surgical margin positivity has long been used for determining the completeness of surgery and estimating patients' prognosis in different types of solid tumors [52, 53, 54, 55, 56]. The NGS‐detected positive resection margins may better characterize minimal residual disease (MRD) of the surgical local margins than the traditional H&E‐based methods. Generally, adjuvant CRT is designed as 2–4 cycles of chemotherapy and 40Gy adjuvant radiotherapy in stage pIIIA/N2 NSCLC patients. However, our findings suggest that adjuvant CRT did not provide additional benefits for patients' prognosis compared to adjuvant CT for patients who received curative‐intent complete surgery with a positive MRD in their surgical margin tissues assessed by broad‐panel NGS assay. Therefore, more intensive treatments such as curative‐intent radiotherapy might be considered for patients who had molecularly positive surgical margins, while other patients could receive regular adjuvant therapy. For example, given that radiotherapy is usually well‐tolerated among stage pIIIA/N2 patients [41, 57], curative‐intent radiotherapy, rather than adjuvant radiotherapy, might be more appropriate and efficient to eliminate molecular levels of MRD. Recently, immunotherapy, particularly immune checkpoint inhibitors (ICI), has shown promising clinical results when included in systematic therapy [58, 59, 60, 61]. Whether postoperative ICI could help control molecular levels of residual disease needs to be further explored in future studies.

A considerable fraction of NGS‐detected positive resection margin was detected in patients who had an H&E‐negative surgical margin. All of the resection margin samples in our study were at least 2.0 cm away from the tumor, which was consistent with previous studies that the bronchial resection margin length of 1.5–2.0 cm was sufficient to achieve tumor‐free bronchus [62, 63]. Also, the possibility of cross‐contamination of tumors and resection margins could be excluded because of no cross‐use of medical devices during the surgical operation. Mutational profiling of surgical margin tissues from R0‐resected patients using the broad‐panel NGS approach demonstrated significant advantages in monitoring molecular levels of residue tumors over the conventional H&E approach, reducing inaccurate identification due to fewer residual tumor cells and inter‐observer variability.

Patients who had driver mutations had indistinguishable DFS compared with those harboring only non‐driver mutations in the resection margin, and both groups of these patients with mutation‐positive resection margins had worse DFS than those with mutation‐negative resection margins. This finding implies that it is important to broadly assess tumor mutations rather than focusing on just a few driver mutations at the surgical margin to fully explore the residual tumor cells. Besides NGS‐detected positive resection margin, we found that some other clinical and molecular factors also had potential prognostic values to estimate recurrence risk in stage pIIIA/N2 NSCLC patients, such as *ERBB2* and *SMARCA4* mutational status and MLNR. Wei *et al*. [64] reported that *ERBB2* mutations were detected in 4.5% of NSCLC cases and *ERBB2* mutation‐positive patients had a lower survival rate. Similarly, *SMARCA4* was usually co‐mutated with *KRAS* in lung adenocarcinoma, and *SMARCA4* mutations have been reported to correlate with adverse clinical outcomes in these patients [65]. Also, multiple groups have shown that stage pIIIA/N2 NSCLC patients with high MLNR had significantly worse survival rates after surgical resection [66, 67]. After considering the effects of these previously identified prognostic factors, we found that NGS‐detected positive resection margin was still a reliable and independent biomarker in stage pIIIA/N2 NSCLC using both multivariable analysis and subgrouping analysis.

## 5 Conclusions

In conclusion, the discovery of NGS‐detected positive margins in R0‐resected stage pIIIA/N2 NSCLC patients successfully confirmed the existence of MRD at the molecular levels, and we used it and other three clinical/molecular factors to construct a COX model that could estimate disease recurrence and patients' prognosis. Examination of microscopically negative margins through NGS provides additional information and represents a valuable approach to identify molecular levels of residue tumors and to evaluate recurrence risk and estimate prognosis.

## Conflict of interest

Jiaohui Pang and Yang Xu are the employees of Nanjing Geneseeq Technology Inc. All remaining authors have declared no conflict of interest.

## Author contributions

JY and SY conceived and supervised the study. LL designed the experiments. LL, KH, TZ, and QY acquired clinical data and performed patient follow‐up. LL, YG, HZ, HS, and ML acquired tissue samples for genomic profiling. LL, YX, and JP performed data analysis. All authors participated in data interpretation. LL wrote the manuscript with the help of all the authors. All authors provided critical revision and consented to the submission of the manuscript.

## Supporting information


**Fig. S1.** The clinical characteristics of the 119 stage IIIA/N2 NSCLC patients.
**Fig. S2.** The tumor samples had a higher number of genetic alterations and variant allele frequency than the corresponding resection margin samples.
**Fig. S3.** The presence of tumor mutations in resection margins was associated with shorter overall survival.
**Fig. S4.** The clinical and molecular features that could serve as potential prognostic biomarkers for stage IIIA/N2 NSCLC patients receiving curative‐intent surgery.
**Fig. S5.** The overall survival of patients receiving different adjuvant treatments.
**Table S1.** The list of the 474 cancer‐related genes analyzed by NGS.
**Table S2.** The 10 signature groups used for the signature analysis.
**Table S3.** The number of different genetic alterations detected in tumor samples of the 119 NSCLC patients.
**Table S4.** The number of different genetic alterations detected in bronchial resection margin samples of the 119 NSCLC patients.
**Table S5.** Genetic variants exclusively identified in bronchial resection margin samples of the 119 NSCLC patients.
**Table S6.** Agreement of detection for tumor‐informed driver alteration‐positive patients using tumor and matched bronchial resection margin samples.
**Table S7.** Comparing the patient clinical characteristics between the two cohorts.
**Table S8.** The univariate analysis of DFS and OS in genes with mutated/CNV frequency higher than 5%.
**Table S9.** The AIC and C‐index of the constructed models using different clinical/molecular factors in both the study group and the validation group.

## Data Availability

The human sequence data generated in this study are not publicly available due to patient privacy requirements but are available upon reasonable request from the corresponding author. Other data generated in this study are available within the article and its supplementary data files.
