# Supplementary material for: Recurrence/prognosis estimation using a molecularly positive surgical margin‐based model calls for alternative curative strategies in pIIIA/N2 NSCLC
Source: Mol Oncol. 2024 Feb 7;18(6):1649–64. doi: 10.1002/1878-0261.13600 (PMC11161728; doi:10.1002/1878-0261.13600)
Supplement: Supplementary file 1 — Fig. S1. The clinical characteristics of the 119 stage IIIA/N2 NSCLC patients. Fig. S2. The tumor samples had a higher number of genetic alterations and variant allele frequency than the corresponding resection margin samples. Fig. S3. The presence of tumor mutations in resection margins was associated with shorter overall survival. Fig. S4. The clinical and molecular features that could serve as potential prognostic biomarkers for stage IIIA/N2 NSCLC patients receiving curative‐intent surgery. Fig. S5. The overall survival of patients receiving different adjuvant treatments. Table S1. The list of the 474 cancer‐related genes analyzed by NGS. Table S2. The 10 signature groups used for the signature analysis. Table S3. The number of different genetic alterations detected in tumor samples of the 119 NSCLC patients. Table S4. The number of different genetic alterations detected in bronchial resection margin samples of the 119 NSCLC patients. Table S5. Genetic variants exclusively identified in bronchial resection margin samples of the 119 NSCLC patients. Table S6. Agreement of detection for tumor‐informed driver alteration‐positive patients using tumor and matched bronchial resection margin samples. Table S7. Comparing the patient clinical characteristics between the two cohorts. Table S8. The univariate analysis of DFS and OS in genes with mutated/CNV frequency higher than 5%. Table S9. The AIC and C‐index of the constructed models using different clinical/molecular factors in both the study group and the validation group. [file MOL2-18-1649-s001.docx]

**Supplementary Tables**

**Supplementary Table 1.** The list of the 474 cancer-related genes analyzed by NGS.

| ADGRB3 | LMO1 | DTL | QKI | BUB1B | NRAS | GNA11 | STAT1 |
| --- | --- | --- | --- | --- | --- | --- | --- |
| AFDN | LRP1B | DUSP2 | RAC1 | CASP8 | NRG1 | GNAQ | STAT3 |
| CCN6 | LYN | EGFR | RAC3 | CBL | NSD1 | GNAS | STK11 |
| EMSY | LZTR1 | EIF1AX | RAD50 | CBLB | NTHL1 | GRIN2A | STMN1 |
| ERBIN | MALT1 | EP300 | RAD51 | CCND1 | NTRK1 | GRM3 | SUFU |
| MRE11 | MAP2K1 | EPAS1 | RAD51B | CCNE1 | NTRK2 | GRM8 | SUMO1 |
| PRKN | MAP2K2 | EPCAM | RAD51C | CD274 | NTRK3 | GSTM1 | TACC3 |
| SEPTIN9 | MAP2K4 | EPHA2 | RAD51D | CD74 | NUTM1 | GSTM4 | TAP1 |
| ABCB1 | MAP3K1 | EPHA3 | RAD54L | CDA | PAK2 | GSTP1 | TAP2 |
| ABCC2 | MAP3K4 | EPHA5 | RAD9A | CDC73 | PAK3 | GSTT1 | TBK1 |
| ABL1 | MAPK1 | ERBB2 | RAF1 | CDH1 | PALB2 | HDAC1 | TEK |
| ADH1B | MAPK3 | ERBB3 | RARA | CDK10 | PALLD | HDAC2 | TEKT4 |
| AIP | MAX | ERBB4 | RARG | CDK12 | PARP1 | HDAC9 | TERC |
| AKT1 | MCL1 | ERCC1 | RASGEF1A | CDK4 | PARP2 | HGF | TERT |
| AKT2 | MDM2 | ERCC2 | RB1 | CDK6 | PAX5 | HLA-A | TET2 |
| AKT3 | MDM4 | ERCC3 | RCC1 | CDK8 | PBRM1 | HMOX1 | TFG |
| ALDH2 | MECOM | ERCC4 | RECQL4 | CDKN1A | PDCD1 | HNF1A | TGFB1 |
| ALK | MED12 | ERCC5 | RELA | CDKN1B | PDCD1LG2 | HNF1B | TGFBR2 |
| AMER1 | MEF2B | ESR1 | RELN | CDKN1C | PDE11A | HRAS | THADA |
| APC | MEN1 | ETV1 | RET | CDKN2A | PDGFRA | HSPB1 | TMEM127 |
| APEX1 | MET | ETV4 | RHOA | CDKN2B | PDGFRB | IDH1 | TMEM167A |
| AR | MGMT | ETV5 | RICTOR | CDKN2C | PDK1 | IDH2 | TMPRSS2 |
| ARAF | MIF | ETV6 | RNF43 | CEBPA | PGR | IFNA6 | TNF |
| ARID1A | MITF | EWSR1 | ROS1 | CEP57 | PHOX2B | IFNB1 | TNFAIP3 |
| ARID1B | MLH1 | EXT1 | RPTOR | CHD4 | PIK3C3 | IFNE | TNFRSF11A |
| ARID2 | MLH3 | EXT2 | RRM1 | CHD8 | PIK3CA | IFNG | TNFRSF14 |
| ARID5B | KMT2A | EZH2 | RUNX1 | CHEK1 | PIK3CD | IFNGR1 | TNFRSF19 |
| ASCL4 | KMT2D | EZR | RUNX1T1 | CHEK2 | PIK3R1 | IFNGR2 | TNFRSF1B |
| ASXL1 | MMP1 | FANCA | SBDS | CREBBP | PIK3R2 | IGF1R | TNFSF11 |
| ATF1 | MPL | FANCC | SDC4 | CRKL | PKHD1 | IGF2 | TOP1 |
| ATIC | MSH2 | FANCD2 | SDHA | CSF1R | PLAG1 | IKBKE | TOP2A |
| ATM | MSH6 | FANCE | SDHB | CTCF | PLCB4 | IKZF1 | TP53 |
| ATR | MTHFR | FANCF | SDHC | CTLA4 | PLK1 | IL13 | TP63 |
| ATRX | MTOR | FANCG | SDHD | CTNNB1 | PMAIP1 | IL1A | TPMT |
| AURKA | MTRR | FANCI | SERPINE1 | CUL3 | PMS1 | IL7R | TSC1 |
| AURKB | MUC5B | FANCL | SETBP1 | CUX1 | PMS2 | INPP4B | TSC2 |
| AXIN2 | MUTYH | FANCM | SETD2 | CXCL8 | PNKP | IRF1 | TSHR |
| AXL | MYC | FAT1 | SF3B1 | CXCR4 | POLD1 | IRF2 | TTF1 |
| B2M | MYCL | FBXW7 | SGK1 | CYLD | POLD3 | ITGB6 | TUBB3 |
| BAD | MYCN | FGF19 | SKP2 | CYP19A1 | POLE | JAK1 | TYMS |
| BAK1 | MYD88 | FGFR1 | SLC34A2 | CYP2A13 | POLH | JAK2 | U2AF1 |
| BAP1 | MYH9 | FGFR2 | SLC3A2 | CYP2A6 | POT1 | JAK3 | UGT1A1 |
| BARD1 | NAT1 | FGFR3 | SMAD2 | CYP2A7 | PPARD | JARID2 | UNG |
| BAX | NBN | FGFR4 | SMAD3 | CYP2B6 | PPP2R1A | JUN | VAMP2 |
| BCL2 | NCOR1 | FH | SMAD4 | CYP2C19 | PRDM1 | KDM5A | VEGFA |
| BCL2L1 | NEIL1 | FLCN | SMAD7 | CYP2C9 | PREX2 | KDR | VHL |
| BCL2L11 | NF1 | FLT1 | SMARCA4 | CYP2D6 | PRF1 | KEAP1 | WAS |
| BCL3 | NF2 | FLT3 | SMARCB1 | CYP3A4 | PRKACA | KIF1B | WRN |
| BCR | NFE2L2 | FLT4 | SMO | CYP3A5 | PRKAR1A | KIT | WT1 |
| BIRC3 | NFKB1 | FOXA1 | SOCS1 | CYSLTR2 | PRKCB | KITLG | XPA |
| BLM | NFKBIA | FOXL2 | SOS1 | DAXX | PRKCI | KLLN | XPC |
| BMPR1A | NKX2-1 | FOXO3 | SOX2 | DDR2 | PRKDC | KMT2A | XRCC1 |
| BRAF | NOS2 | FOXP1 | SPOP | DENND1A | PRSS1 | KMT2B | XRCC2 |
| BRCA1 | NOS3 | FRG1 | SPRED1 | DHFR | PRSS3 | KMT2C | XRCC3 |
| BRCA2 | NOTCH1 | GATA1 | SPRY4 | DICER1 | PTCH1 | KMT2D | XRCC4 |
| BRD4 | NOTCH2 | GATA2 | SRC | DLL3 | PTEN | KRAS | XRCC5 |
| BRIP1 | NOTCH3 | GATA3 | SRSF2 | DNMT3A | PTK2 | LHCGR | YAP1 |
| BTG2 | NPM1 | GATA4 | SRY | DOT1L | PTPN11 | LIG3 | ZNF2 |
| BTK | NQO1 | GATA6 | STAG2 | DPYD | PTPN13 | LIG4 | ZNF217 |
| LIN28B | ZNF703 |  |  |  |  |  |  |

**Supplementary Table 2.** The 10 signature groups used for the signature analysis.

| **Mutation signature catalog** | **Description** |
| --- | --- |
| Age | COSMIC_1 |
| APOBEC | COSMIC_2+ COSMIC_13 |
| BRCA | COSMIC_3 |
| Smoking | COSMIC_4 |
| dMMR | COSMIC_6 + COSMIC_15 + COSMIC_20 + COSMIC_26 |
| Ultraviolet | COSMIC_7 |
| Immunoglobulin | COSMIC_9 |
| POLE | COSMIC_10 |
| Temozolomide | COSMIC_11 |
| Others | 1- Age - APOBEC - BRCA - Smoking - MMRdeficiency - Ultraviolet - Immunoglobulin - POLE - Temozolomide |

**Supplementary Table 3.** The number of different genetic alterations detected in tumor samples of the 119 NSCLC patients.

| **Patient ID** | **Fusion** | **CNV (amplification)** | **CNV (deletion)** | **SNV** | **Indel** | **Max VAF** | **Total** |
| --- | --- | --- | --- | --- | --- | --- | --- |
| P001 | 1 | 0 | 0 | 4 | 0 | 49.03% | 5 |
| P002 | 0 | 0 | 1 | 8 | 0 | 30.94% | 9 |
| P003 | 0 | 1 | 0 | 10 | 2 | 27.05% | 13 |
| P004 | 1 | 0 | 0 | 2 | 0 | 15.72% | 3 |
| P005 | 0 | 0 | 0 | 10 | 0 | 12.00% | 10 |
| P006 | 0 | 0 | 0 | 8 | 3 | 55.01% | 11 |
| P007 | 0 | 0 | 0 | 1 | 2 | 47.75% | 3 |
| P008 | 0 | 2 | 0 | 10 | 2 | 39.18% | 14 |
| P009 | 1 | 1 | 0 | 5 | 1 | 43.54% | 8 |
| P010 | 0 | 0 | 0 | 6 | 1 | 12.11% | 7 |
| P011 | 0 | 1 | 0 | 4 | 0 | 40.28% | 5 |
| P012 | 0 | 0 | 0 | 1 | 2 | 55.75% | 3 |
| P013 | 0 | 2 | 2 | 5 | 1 | 67.51% | 10 |
| P014 | 0 | 0 | 0 | 1 | 1 | 24.29% | 2 |
| P015 | 0 | 2 | 1 | 5 | 1 | 40.17% | 9 |
| P016 | 0 | 0 | 0 | 4 | 2 | 32.22% | 6 |
| P017 | 0 | 2 | 0 | 8 | 1 | 59.23% | 11 |
| P018 | 0 | 0 | 0 | 3 | 1 | 32.11% | 4 |
| P019 | 0 | 4 | 0 | 5 | 1 | 60.09% | 10 |
| P020 | 0 | 1 | 0 | 7 | 0 | 31.57% | 8 |
| P021 | 0 | 0 | 0 | 17 | 0 | 19.41% | 17 |
| P022 | 0 | 0 | 0 | 8 | 1 | 24.40% | 9 |
| P023 | 0 | 0 | 0 | 15 | 1 | 77.65% | 16 |
| P024 | 0 | 1 | 0 | 2 | 2 | 34.35% | 5 |
| P025 | 0 | 0 | 0 | 6 | 0 | 45.29% | 6 |
| P026 | 0 | 1 | 0 | 8 | 1 | 70.93% | 10 |
| P027 | 0 | 0 | 0 | 4 | 3 | 33.88% | 7 |
| P028 | 0 | 0 | 0 | 8 | 2 | 81.76% | 10 |
| P029 | 1 | 0 | 0 | 24 | 0 | 35.37% | 25 |
| P030 | 0 | 0 | 0 | 6 | 2 | 53.52% | 8 |
| P031 | 0 | 2 | 1 | 11 | 0 | 22.73% | 14 |
| P032 | 0 | 0 | 0 | 15 | 1 | 44.64% | 16 |
| P033 | 0 | 1 | 0 | 3 | 1 | 19.57% | 5 |
| P034 | 0 | 0 | 0 | 2 | 3 | 23.20% | 5 |
| P035 | 0 | 0 | 0 | 1 | 0 | 8.43% | 1 |
| P036 | 0 | 0 | 0 | 3 | 1 | 23.79% | 4 |
| P037 | 1 | 1 | 0 | 0 | 0 | 0.82% | 2 |
| P038 | 0 | 0 | 0 | 2 | 1 | 17.45% | 3 |
| P039 | 0 | 0 | 0 | 0 | 0 | 0 | 0 |
| P040 | 0 | 6 | 1 | 5 | 3 | 75.43% | 15 |
| P041 | 0 | 1 | 0 | 10 | 2 | 57.74% | 13 |
| P042 | 0 | 0 | 0 | 8 | 0 | 28.48% | 8 |
| P043 | 0 | 2 | 1 | 5 | 0 | 77.91% | 8 |
| P044 | 1 | 0 | 0 | 4 | 1 | 24.99% | 6 |
| P047 | 0 | 0 | 0 | 7 | 1 | 50.72% | 8 |
| P048 | 1 | 2 | 0 | 45 | 5 | 67.45% | 53 |
| P049 | 0 | 1 | 0 | 5 | 0 | 89.55% | 6 |
| P050 | 1 | 0 | 0 | 3 | 1 | 8.01% | 5 |
| P051 | 0 | 0 | 0 | 13 | 1 | 19.64% | 14 |
| P052 | 2 | 0 | 0 | 1 | 1 | 38.08% | 4 |
| P053 | 0 | 0 | 0 | 3 | 2 | 35.98% | 5 |
| P054 | 0 | 0 | 2 | 11 | 1 | 29.10% | 14 |
| P055 | 0 | 3 | 0 | 2 | 0 | 5.30% | 5 |
| P056 | 0 | 0 | 0 | 18 | 2 | 34.19% | 20 |
| P057 | 0 | 0 | 0 | 13 | 1 | 44.29% | 14 |
| P058 | 0 | 0 | 0 | 19 | 5 | 69.79% | 24 |
| P059 | 0 | 0 | 0 | 16 | 1 | 40.39% | 17 |
| P060 | 0 | 0 | 1 | 1 | 0 | 64.95% | 2 |
| P061 | 0 | 0 | 0 | 1 | 1 | 22.71% | 2 |
| P062 | 0 | 0 | 0 | 2 | 2 | 16.59% | 4 |
| P063 | 0 | 0 | 0 | 11 | 0 | 59.79% | 11 |
| P064 | 0 | 2 | 0 | 6 | 1 | 75.73% | 9 |
| P066 | 1 | 0 | 0 | 1 | 0 | 13.15% | 2 |
| P067 | 0 | 0 | 0 | 4 | 1 | 57.46% | 5 |
| P068 | 0 | 0 | 0 | 8 | 2 | 19.37% | 10 |
| P069 | 1 | 0 | 0 | 3 | 1 | 50.42% | 5 |
| P070 | 0 | 0 | 0 | 10 | 1 | 39.17% | 11 |
| P071 | 1 | 0 | 0 | 1 | 0 | 11.21% | 2 |
| P072 | 0 | 0 | 0 | 8 | 2 | 19.81% | 10 |
| P073 | 0 | 4 | 0 | 15 | 1 | 78.02% | 20 |
| P074 | 0 | 0 | 0 | 2 | 3 | 62.01% | 5 |
| P075 | 0 | 0 | 0 | 2 | 0 | 3.49% | 2 |
| P076 | 0 | 2 | 0 | 22 | 2 | 63.45% | 26 |
| P077 | 1 | 0 | 0 | 1 | 0 | 12.64% | 2 |
| P078 | 1 | 1 | 0 | 2 | 2 | 18.41% | 6 |
| P079 | 0 | 0 | 0 | 2 | 0 | 20.76% | 2 |
| P080 | 0 | 3 | 1 | 23 | 3 | 82.07% | 30 |
| P081 | 0 | 0 | 0 | 6 | 1 | 63.82% | 7 |
| P082 | 0 | 0 | 0 | 11 | 0 | 62.50% | 11 |
| P084 | 1 | 0 | 0 | 6 | 1 | 32.69% | 8 |
| P085 | 0 | 0 | 2 | 5 | 2 | 30.37% | 9 |
| P086 | 1 | 1 | 0 | 1 | 0 | 100.00% | 3 |
| P087 | 0 | 0 | 0 | 6 | 1 | 61.24% | 7 |
| P088 | 0 | 0 | 0 | 17 | 2 | 56.01% | 19 |
| P089 | 0 | 0 | 0 | 8 | 2 | 30.47% | 10 |
| P090 | 0 | 0 | 0 | 10 | 3 | 21.01% | 13 |
| P091 | 0 | 1 | 0 | 3 | 1 | 36.44% | 5 |
| P092 | 0 | 0 | 0 | 5 | 1 | 22.96% | 6 |
| P093 | 0 | 0 | 0 | 3 | 0 | 28.66% | 3 |
| P094 | 0 | 0 | 0 | 5 | 2 | 24.90% | 7 |
| P095 | 0 | 3 | 0 | 11 | 1 | 78.48% | 15 |
| P096 | 0 | 0 | 0 | 11 | 3 | 29.44% | 14 |
| P097 | 0 | 0 | 0 | 1 | 0 | 14.66% | 1 |
| P098 | 2 | 2 | 2 | 4 | 0 | 74.22% | 10 |
| P099 | 1 | 0 | 0 | 1 | 1 | 29.17% | 3 |
| P101 | 0 | 0 | 0 | 15 | 1 | 68.29% | 16 |
| P102 | 2 | 1 | 1 | 2 | 1 | 41.67% | 7 |
| P103 | 0 | 4 | 2 | 9 | 1 | 24.26% | 16 |
| P104 | 0 | 0 | 0 | 0 | 0 | 0 | 0 |
| P105 | 1 | 2 | 1 | 6 | 2 | 43.09% | 12 |
| P108 | 0 | 0 | 2 | 11 | 3 | 62.55% | 16 |
| P109 | 0 | 0 | 0 | 13 | 0 | 74.07% | 13 |
| P110 | 0 | 0 | 0 | 15 | 2 | 67.55% | 17 |
| P111 | 0 | 0 | 0 | 2 | 2 | 11.37% | 4 |
| P112 | 0 | 0 | 0 | 13 | 1 | 33.51% | 14 |
| P113 | 0 | 0 | 0 | 3 | 0 | 11.64% | 3 |
| P114 | 0 | 0 | 0 | 20 | 2 | 35.77% | 22 |
| P115 | 0 | 0 | 0 | 10 | 1 | 82.17% | 11 |
| P116 | 2 | 1 | 0 | 6 | 0 | 41.12% | 9 |
| P117 | 0 | 0 | 0 | 18 | 1 | 40.87% | 19 |
| P118 | 0 | 3 | 0 | 13 | 0 | 55.82% | 16 |
| P119 | 0 | 2 | 1 | 14 | 1 | 37.38% | 18 |
| P120 | 0 | 0 | 0 | 6 | 0 | 45.45% | 6 |
| P121 | 0 | 7 | 1 | 4 | 1 | 85.73% | 13 |
| P122 | 0 | 0 | 0 | 9 | 4 | 32.72% | 13 |
| P125 | 1 | 2 | 0 | 8 | 1 | 50.55% | 12 |
| P126 | 0 | 0 | 0 | 21 | 2 | 56.40% | 23 |
| P127 | 0 | 0 | 0 | 10 | 2 | 47.51% | 12 |
| P128 | 0 | 0 | 0 | 17 | 4 | 30.30% | 21 |
| **Total** | 26 | 78 | 23 | 914 | 142 | - | 1183 |

**Supplementary Table 4.** The number of different genetic alterations detected in bronchial resection margin samples of the 119 NSCLC patients.

| **Patient ID** | **Fusion** | **Indel** | **SNV** | **Max VAF** | **Total** |
| --- | --- | --- | --- | --- | --- |
| P008 | 0 | 1 | 3 | 8.05% | 4 |
| P011 | 0 | 0 | 3 | 22.20% | 3 |
| P012 | 0 | 1 | 1 | 2.61% | 2 |
| P013 | 0 | 1 | 2 | 2.96% | 3 |
| P017 | 0 | 1 | 3 | 26.65% | 4 |
| P018 | 0 | 0 | 2 | 6.82% | 2 |
| P019 | 0 | 1 | 5 | 58.50% | 6 |
| P023 | 0 | 0 | 1 | 0.84% | 1 |
| P024 | 0 | 1 | 1 | 0.45% | 2 |
| P026 | 0 | 0 | 1 | 3.53% | 1 |
| P029 | 0 | 0 | 3 | 7.06% | 3 |
| P032 | 0 | 1 | 3 | 11.72% | 4 |
| P037 | 1 | 0 | 0 | 0.58% | 1 |
| P043 | 0 | 0 | 4 | 29.06% | 4 |
| P044 | 0 | 0 | 1 | 0.42% | 1 |
| P048 | 1 | 1 | 34 | 24.14% | 36 |
| P049 | 0 | 0 | 1 | 10.41% | 1 |
| P050 | 1 | 1 | 1 | 2.96% | 3 |
| P051 | 0 | 0 | 1 | 2.46% | 1 |
| P053 | 0 | 1 | 2 | 3.48% | 3 |
| P056 | 0 | 2 | 8 | 6.33% | 10 |
| P057 | 0 | 1 | 4 | 3.05% | 5 |
| P058 | 0 | 0 | 1 | 1.25% | 1 |
| P059 | 0 | 0 | 10 | 7.18% | 10 |
| P060 | 0 | 0 | 1 | 8.23% | 1 |
| P062 | 0 | 1 | 0 | 0.10% | 1 |
| P063 | 0 | 0 | 2 | 6.72% | 2 |
| P064 | 0 | 1 | 4 | 28.44% | 5 |
| P067 | 0 | 1 | 1 | 1.68% | 2 |
| P069 | 0 | 0 | 1 | 0.25% | 1 |
| P072 | 0 | 0 | 2 | 10.31% | 2 |
| P076 | 0 | 0 | 2 | 1.12% | 2 |
| P078 | 1 | 2 | 2 | 4.62% | 5 |
| P084 | 0 | 0 | 1 | 0.90% | 1 |
| P087 | 0 | 1 | 6 | 1.34% | 7 |
| P088 | 0 | 2 | 5 | 18.50% | 7 |
| P090 | 0 | 1 | 0 | 0.53% | 1 |
| P092 | 0 | 1 | 1 | 2.34% | 2 |
| P095 | 0 | 1 | 2 | 8.50% | 3 |
| P096 | 0 | 3 | 5 | 17.92% | 8 |
| P098 | 0 | 0 | 1 | 9.47% | 1 |
| P101 | 0 | 0 | 3 | 44.65% | 3 |
| P103 | 0 | 1 | 8 | 12.05% | 9 |
| P108 | 0 | 1 | 4 | 7.95% | 5 |
| P110 | 0 | 0 | 9 | 4.60% | 9 |
| P112 | 0 | 0 | 3 | 1.30% | 3 |
| P113 | 0 | 0 | 1 | 10.55% | 1 |
| P115 | 0 | 1 | 1 | 6.22% | 2 |
| P116 | 1 | 0 | 5 | 33.51% | 6 |
| P117 | 0 | 0 | 1 | 3.17% | 1 |
| P118 | 0 | 0 | 13 | 14.33% | 13 |
| P119 | 0 | 1 | 9 | 21.28% | 10 |
| P121 | 0 | 0 | 1 | 1.20% | 1 |
| P126 | 0 | 1 | 7 | 11.88% | 8 |
| P127 | 0 | 0 | 2 | 3.31% | 2 |
| P128 | 0 | 1 | 11 | 5.26% | 12 |
| **Total** | 5 | 33 | 209 | - | 247 |

**Supplementary Table 5.** Genetic variants exclusively identified in bronchial resection margin samples of the 119 NSCLC patients.

| **ID** | **Gene** | **AA change** | **Variant type** | **Cosmic ID** | **OncoKB** |
| --- | --- | --- | --- | --- | --- |
| P043 | ATM | p.V3028I | Missense mutation | ~ | Unknown |
| P080 | BCL3 | ~ | Amplification | ~ | Unknown |
| P057 | CD274 | p.D122V | Missense mutation | ~ | Unknown |
| P080 | CDK4 | ~ | Amplification | ~ | Level 4 |
| P011 | CDK6 | p.D268H | Missense mutation | ~ | Unknown |
| P006 | CHEK2 | p.A290T | Missense mutation | COSM1033068 | Unknown |
| P048 | FAT1 | p.R1953T | Missense mutation | ~ | Unknown |
| P126 | FGFR4 | p.D427H | Missense mutation | ~ | Unknown |
| P064 | GNAS | p.G187A | Missense mutation | ~ | Unknown |
| P039 | GRIN2A | p.W609* | Nonsense mutation | ~ | Unknown |
| P101 | IGF1R | p.W14C | Missense mutation | ~ | Unknown |
| P108 | INPP4B | p.Y239C | Missense mutation | ~ | Unknown |
| P011 | KDM5A | p.P1474Tfs*3 | Frame shift deletion | ~ | Unknown |
| P029 | KEAP1 | p.R470C | Missense mutation | COSM564847 | Unknown |
| P037 | MYC | ~ | Amplification | ~ | Unknown |
| P032 | MYCL | p.S68L | Missense mutation | COSM9793782 | Unknown |
| P048 | PDGFRB | p.T262I | Missense mutation | ~ | Unknown |
| P043 | PIK3C3 | p.Q645E | Missense mutation | ~ | Unknown |
| P085 | PIK3CA | p.H1047R | Missense mutation | COSM775 | Level 1; Level 4 |
| P102 | PLCB4 | p.P654T | Missense mutation | COSM1262364 | Unknown |
| P032 | POLE | p.E450K | Missense mutation | ~ | Unknown |
| P096 | SMARCA4 | ~ | Splice variant | ~ | Unknown |
| P048 | SOCS1 | p.E142K | Missense mutation | COSM6906630 | Unknown |
| P064 | THADA | p.K92N | Missense mutation | ~ | Unknown |
| P117 | XRCC3 | p.R302C | Missense mutation | ~ | Unknown |

**Supplementary Table 6.** Agreement of detection for tumor-informed driver alteration-positive patients using tumor and matched bronchial resection margin samples.

|  | # Tumors positive for driver alterations | # Margins positive for driver alterations | Agreement (%) |
| --- | --- | --- | --- |
| Study cohort (n=100) | 94 | 37 | 39.4% |
| Validation cohort (n=19) | 19 | 10 | 52.6% |
| Total patients (n=119) | 113 | 47 | 41.6% |

**Supplementary Table 7.** Comparing the patient clinical characteristics between the two cohorts.

| **Characteristics** | **Study cohort** | **Validation cohort** | ***P*-value** |
| --- | --- | --- | --- |
|  | **(n=100)** | **(n=19)** |  |
| **Median follow-up (months)**  **[median (95% CI)]** | 38.2 (34.0-47.5) | 30.0 (16.1-NA) |  |
| **Median age (years)**  **[median (range)]** | 60 (39-77) | 60 (47-73) | 0.761 |
| **Sex** |  |  | 0.196 |
| Female | 38 (38.0%) | 4 (21.1%) |  |
| Male | 62 (62.0%) | 15 (78.9%) |  |
| **Smoking history** |  |  | 0.214 |
| Yes | 46 (46.0%) | 12 (63.2%) |  |
| No | 54 (54.0%) | 7 (36.8%) |  |
| **Pathological type** |  |  | 0.020 |
| Adenocarcinoma | 64 (64.0%) | 6 (31.6%) |  |
| Squamous carcinoma | 28 (28.0%) | 10 (52.6%) |  |
| Others | 8 (8.0%) | 3 (15.8%) |  |
| **Operation procedure** |  |  | 0.410 |
| Pneumonectomy | 2 (2.0%) | 1 (5.3%) |  |
| Lobectomy/Segmentectomy | 98 (98.0%) | 18 (94.7%) |  |
| **Adjuvant therapy** |  |  | 0.583 |
| Chemotherapy | 69 (69.0%) | 15 (78.9%) |  |
| Chemoradiotherapy | 31 (31.0%) | 4 (21.1%) |  |
| **Tumor location** |  |  | 0.437 |
| Left-sided | 39 (39.0%) | 5 (26.3%) |  |
| Right-sided | 61 (61.0%) | 14 (73.7%) |  |
| **Performance status (KPS)**  **[median (range)]** | 90 (60-100) | 90 (80-90) | 0.430 |
| **Number of examined lymph nodes**  **[median (range)]** | 16 (4-53) | 21 (9-45) | 0.076 |
| **Number of positive lymph nodes**  **[median (range)]** | 3 (1-23) | 4 (1-18) | 0.886 |
| **Metastatic lymph node ratio (MLNR)**  **[median (range)]** | 23.1% (1.9-100) | 25.0% (2.2-76.9) | 0.688 |

**Supplementary Table 8.** The univariate analysis of DFS and OS in genes with mutated/CNV frequency higher than 5%.

| **Gene** | **Frequency** | **HR (95% CI)**  **for DFS** | ***P* value (DFS)** | **HR (95% CI)**  **for OS** | ***P* value (OS)** |
| --- | --- | --- | --- | --- | --- |
| SMARCA4_mut | 0.05 | 4.92 (1.89~12.78) | <0.001 | 5.13 (1.11~23.72) | 0.020 |
| NOS2_mut | 0.06 | 3.24 (1.27~8.25) | 0.009 | 5.61 (1.88~16.71) | <0.001 |
| ERBB2_mut | 0.05 | 2.81 (0.99~7.94) | 0.042 | 7.22 (2.04~25.53) | <0.001 |
| TP53_mut | 0.72 | 1.86 (0.99~3.5) | 0.051 | 3.42 (1.02~11.47) | 0.035 |
| ABCB1_mut | 0.07 | 0.18 (0.03~1.33) | 0.059 | 0.43 (0.06~3.23) | 0.402 |
| GRIN2A_mut | 0.06 | 0.43 (0.1~1.75) | 0.223 | 0 (0~Inf) | 0.178 |
| APC_mut | 0.07 | 1.73 (0.69~4.35) | 0.239 | 1 (0.23~4.24) | 0.995 |
| PIK3CA_mut | 0.13 | 1.51 (0.74~3.09) | 0.252 | 1.32 (0.38~4.55) | 0.656 |
| LRP1B_mut | 0.14 | 0.66 (0.3~1.47) | 0.307 | 0.17 (0.02~1.27) | 0.050 |
| MCL1_CNV | 0.12 | 1.43 (0.7~2.92) | 0.324 | 1.34 (0.4~4.51) | 0.633 |
| EGFR_CNV | 0.07 | 1.52 (0.65~3.56) | 0.327 | 0.65 (0.09~4.83) | 0.672 |
| CREBBP_mut | 0.05 | 0.5 (0.12~2.06) | 0.330 | 0.77 (0.1~5.71) | 0.798 |
| FLT4_mut | 0.1 | 1.5 (0.64~3.5) | 0.342 | 0.5 (0.07~3.73) | 0.492 |
| NTRK3_mut | 0.05 | 1.55 (0.56~4.28) | 0.397 | 1.84 (0.43~7.86) | 0.402 |
| CDKN2A_mut | 0.12 | 0.72 (0.33~1.58) | 0.409 | 0.55 (0.13~2.32) | 0.406 |
| KEAP1_mut | 0.09 | 1.42 (0.61~3.32) | 0.418 | 1.14 (0.27~4.86) | 0.860 |
| SETD2_mut | 0.06 | 1.45 (0.52~4.02) | 0.473 | 2.03 (0.47~8.7) | 0.329 |
| CHD8_mut | 0.05 | 1.44 (0.45~4.63) | 0.538 | 1.31 (0.17~9.9) | 0.793 |
| KDR_mut | 0.06 | 0.71 (0.22~2.26) | 0.558 | 1.07 (0.25~4.57) | 0.929 |
| CDKN2B_CNV | 0.05 | 0.72 (0.22~2.3) | 0.574 | 0.66 (0.09~4.87) | 0.679 |
| ROS1_mut | 0.06 | 1.3 (0.47~3.61) | 0.608 | 0 (0~Inf) | 0.247 |
| CDKN2A_CNV | 0.08 | 0.81 (0.32~2.03) | 0.655 | 0.42(0.06~3.12) | 0.381 |
| NSD1_mut | 0.07 | 1.24 (0.45~3.45) | 0.675 | 0 (0~Inf) | 0.193 |
| PREX2_mut | 0.05 | 1.24 (0.44~3.48) | 0.686 | 0.63 (0.08~4.74) | 0.650 |
| NFE2L2_mut | 0.07 | 1.19 (0.47~2.97) | 0.716 | 3.48 (1.16~10.42) | 0.017 |
| BRCA2_mut | 0.06 | 0.81 (0.25~2.6) | 0.729 | 1.95 (0.58~6.57) | 0.270 |
| ARID1B_mut | 0.05 | 1.2 (0.43~3.3) | 0.730 | 1.45 (0.34~6.17) | 0.615 |
| EGFR_mut | 0.49 | 0.92 (0.56~1.53) | 0.749 | 1.06 (0.48~2.33) | 0.894 |
| FAT1_mut | 0.06 | 0.85 (0.27~2.73) | 0.787 | 0.9 (0.12~6.68) | 0.915 |
| RB1_mut | 0.08 | 1.12 (0.48~2.61) | 0.792 | 0.52 (0.07~3.85) | 0.514 |
| EP300_mut | 0.08 | 1.12 (0.4~3.1) | 0.829 | 0.57 (0.08~4.21) | 0.573 |
| PKHD1_mut | 0.1 | 1.08 (0.46~2.52) | 0.858 | 0.4 (0.05~2.93) | 0.346 |
| ATM_mut | 0.05 | 1.07 (0.39~2.94) | 0.903 | 0 (0~Inf) | 0.217 |
| SETBP1_mut | 0.05 | 0.93 (0.29~2.99) | 0.904 | 1.8 (0.42~7.78) | 0.425 |
| EPHA3_mut | 0.08 | 0.95 (0.38~2.37) | 0.911 | 0 (0~Inf) | 0.112 |
| NOTCH1_mut | 0.05 | 0.95 (0.3~3.04) | 0.929 | 1.43 (0.34~6.09) | 0.627 |
| ALK_mut | 0.05 | 1.04 (0.33~3.33) | 0.947 | 1.34 (0.31~5.74) | 0.691 |

mut: mutation; CNV: copy number variation; DFS: disease-free survival; OS: overall survival.

**Supplementary Table 9.** The AIC and C-index of the constructed models using different clinical/molecular factors in both the study group and the validation group.

| **Factor(s) used in the model** | **Study group** | |  | **Validation** |
| --- | --- | --- | --- | --- |
|  | **AIC** | **C-index** |  | **C-index** |
| Margin (NGS)+MLNR+ERBB2+SMARCA4 | 466.09 | 0.679 |  | 0.6352 |
| MLNR+ERBB2+SMARCA4 | 472.1 | 0.608 |  | 0.6066 |
| Margin (NGS)+ERBB2+SMARCA4 | 467.08 | 0.6638 |  | 0.6066 |
| Margin (NGS)+MLNR+SMARCA4 | 468.21 | 0.6661 |  | 0.6311 |
| Margin (NGS)+MLNR+ERBB2 | 470.96 | 0.6428 |  | 0.6352 |

**Supplementary Figures**


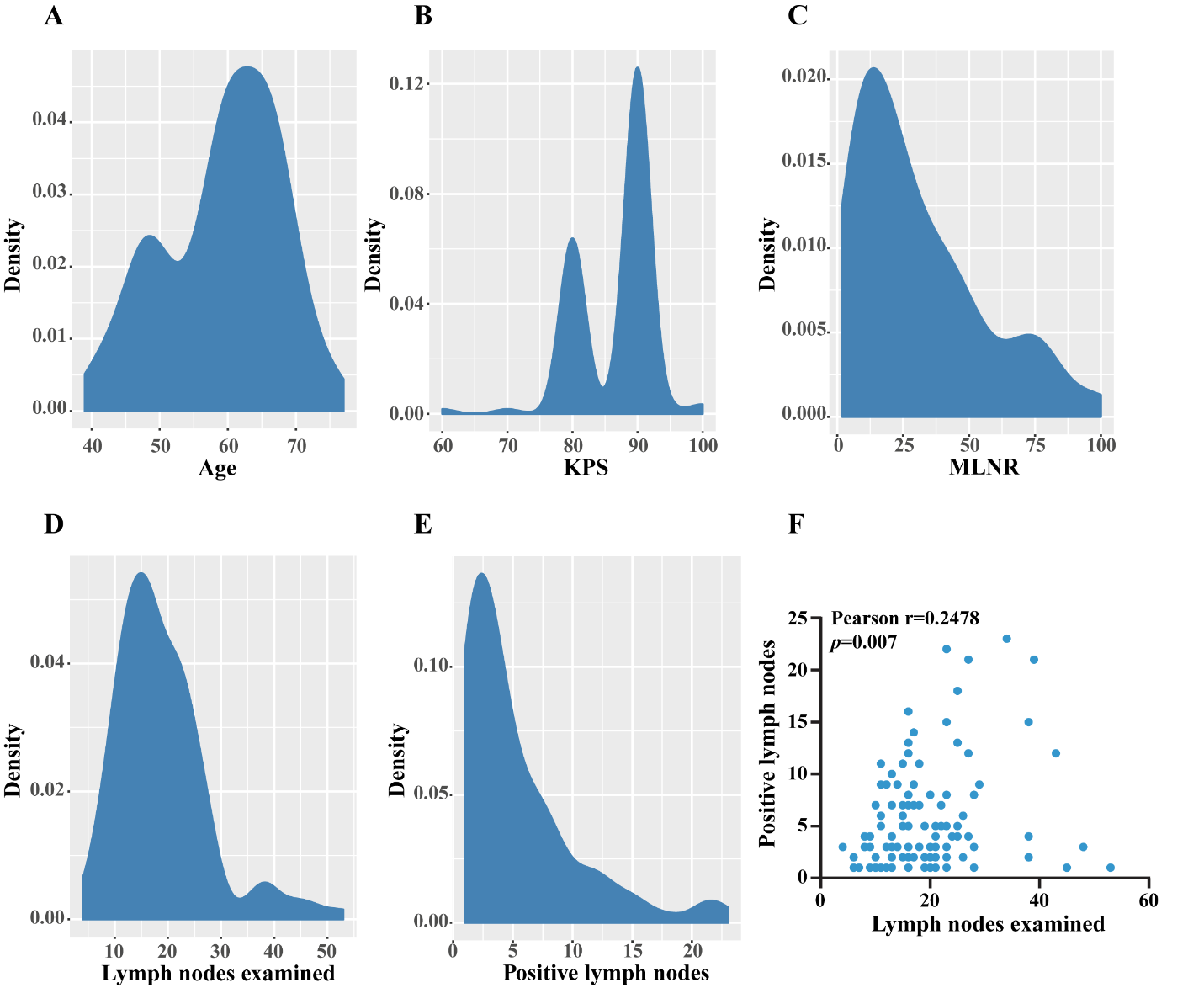


**Supplementary Figure 1.** The clinical characteristics of the 119 stage IIIA/N2 NSCLC patients. (**A**-**E**) The density plot of age (**A**), KPS (**B**), MLNR (**C**), the number of examined lymph nodes (**D**), and the number of positive lymph nodes (**E**). **(F)** The scatterplot of the number of examined lymph nodes and the number of positive lymph nodes. KPS, Karnofsky Performance Status; MLNR, metastatic lymph node ratio.


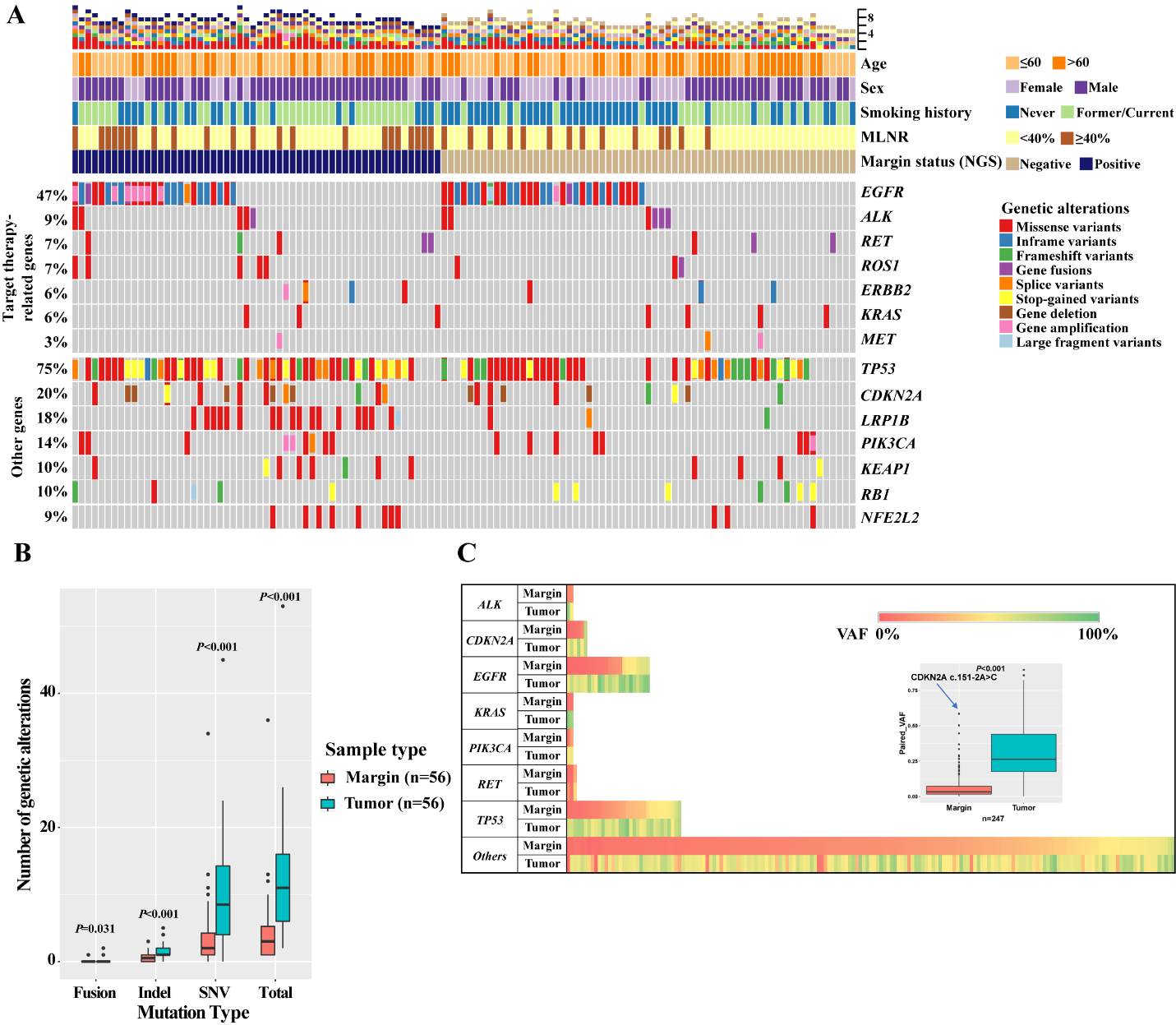


**Supplementary Figure 2.** The tumor samples had a higher number of genetic alterations and variant allele frequency than the corresponding resection margin samples. (**A**) The genetic profile of the top 14 mutated genes identified in the 119 stage IIIA/N2 NSCLC patients. The corresponding clinical characteristics of the patients were listed above the oncoprint plot. (**B**) The number of different types of genetic alterations when comparing the tumor and resection margin samples. The 56 resection margin samples that were classified as NGS-detected positive resection margins and the paired tumor samples were used for the analysis. (**C**) The comparison of variant allele frequency of different mutations for each patient. The 56 resection margin samples that were classified as NGS-detected positive resection margins and the paired tumor samples were used for the analysis. SNV, single nucleotide variation; VAF, variant allele frequency.


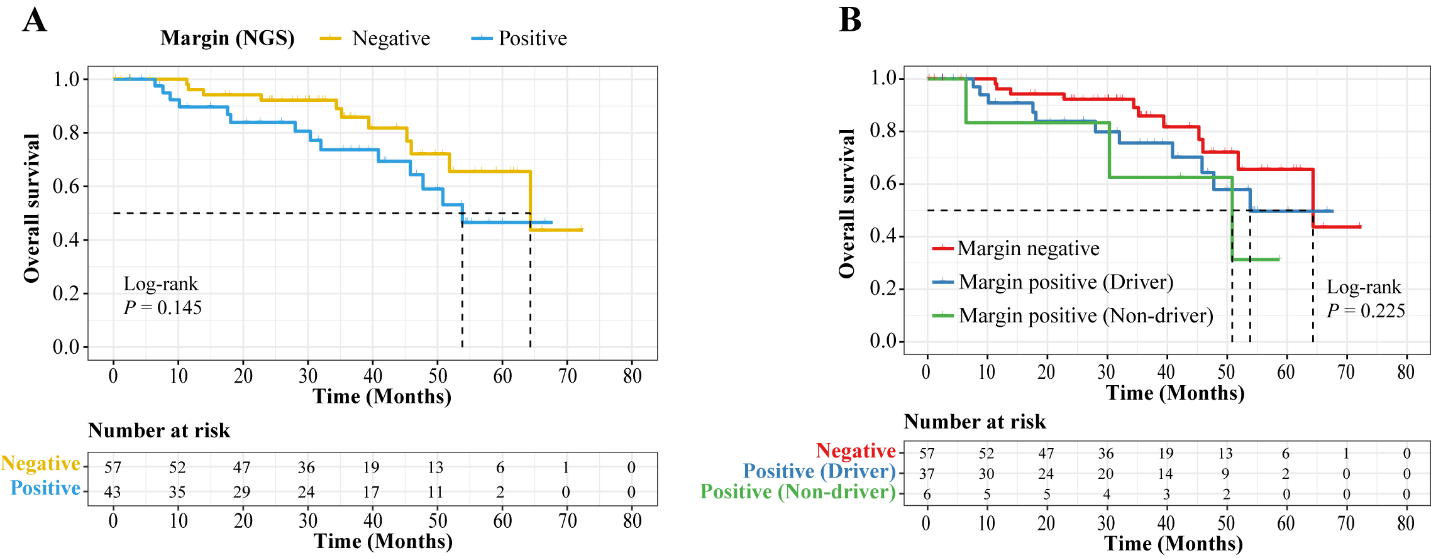


**Supplementary Figure 3.** The presence of tumor mutations in resection margins was associated with shorter overall survival. **(A)** Kaplan-Meier curve of overall survival in 100 stage IIIA/N2 NSCLC patients (study cohort) in strata of the presence or absence of tumor mutations in resection margin. The estimated median survival time is represented by the location on the x-axis where the curve intersects with the horizontal dashed line drawn at a 50% survival probability. **(B)** Kaplan-Meier curve of overall survival in 100 stage IIIA/N2 NSCLC patients (study cohort) in strata of the resection margin mutational status (i.e., no mutations, with only non-driver mutations, or with lung cancer driver mutations). The estimated median survival time is represented by the location on the x-axis where the curve intersects with the horizontal dashed line drawn at a 50% survival probability.


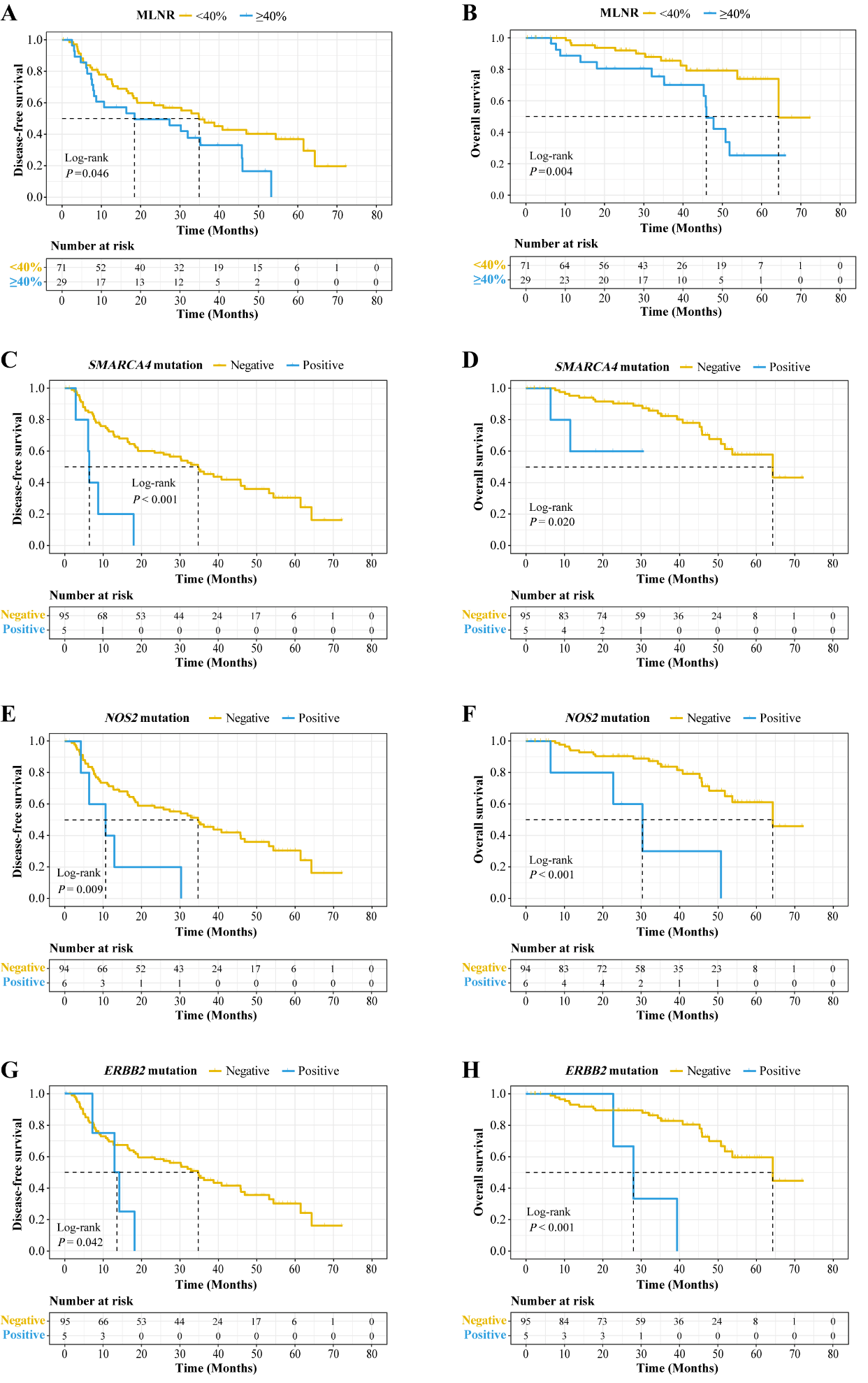


**Supplementary Figure 4.** The clinical and molecular features that could serve as potential prognostic biomarkers for stage IIIA/N2 NSCLC patients receiving curative-intent surgery. (**A-B**) Kaplan-Meier curve of DFS (**A**) or OS (**B**) in 100 stage IIIA/N2 NSCLC patients in strata of the MLNR percentage. (**C-D**) Kaplan-Meier curve of DFS (**C**) or OS (**D**) in 100 stage IIIA/N2 NSCLC patients in strata of *SMARCA4* mutation status. (**E-F**) Kaplan-Meier curve of DFS (**E**) or OS (**F**) in 100 stage IIIA/N2 NSCLC patients in strata of *NOS2* mutation status. (**G-H**) Kaplan-Meier curve of DFS (**G**) or OS (**H**) in 100 stage IIIA/N2 NSCLC patients in strata of *ERBB2* mutation status. The estimated median survival time is represented by the location on the x-axis where the curve intersects with the horizontal dashed line drawn at a 50% survival probability.


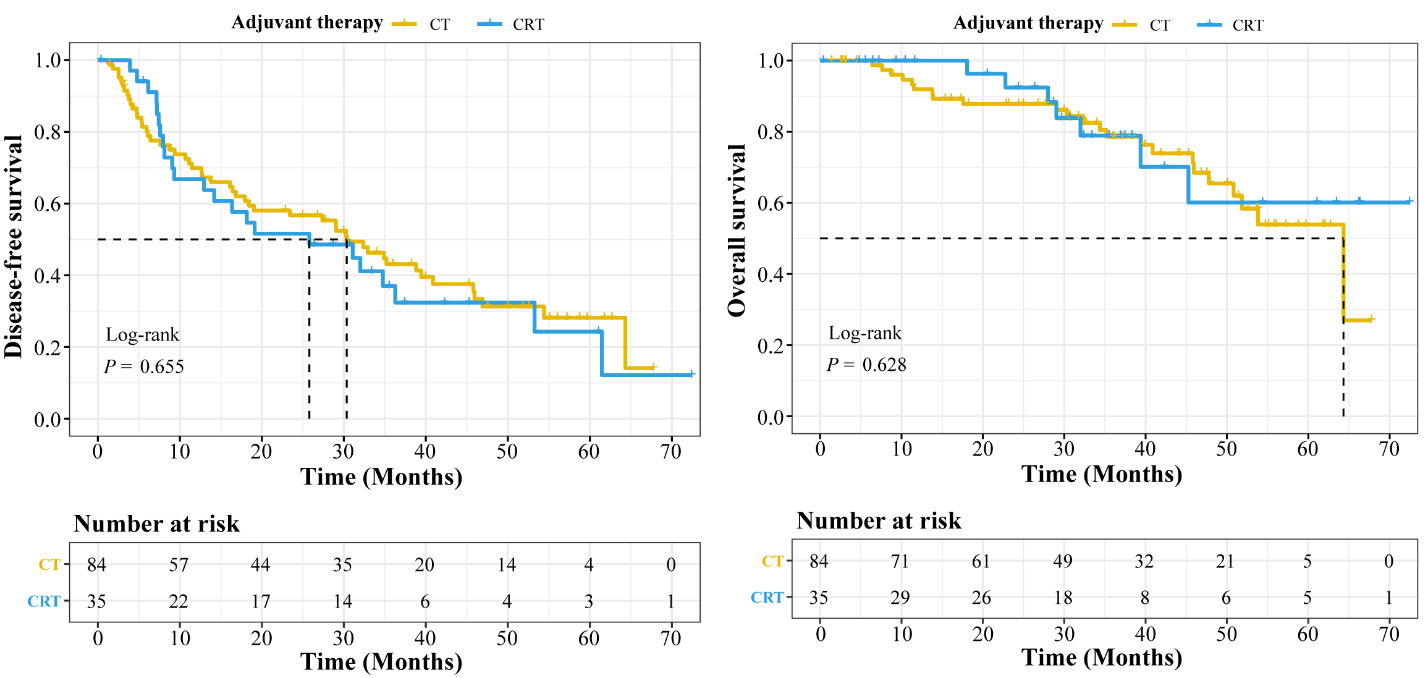


**Supplementary Figure 5.** The overall survival of patients receiving different adjuvant treatments. (**A, B**) Kaplan-Meier curve of disease-free survival (**A**) and overall survival (**B**) in 119 stage IIIA/N2 NSCLC patients in strata of the adjuvant treatment regimen. The estimated median survival time is represented by the location on the x-axis where the curve intersects with the horizontal dashed line drawn at a 50% survival probability. CT, chemotherapy; CRT, chemoradiotherapy.
